# Triglyceride-glucose index for the detection of subclinical heart failure with preserved ejection fraction in patients with type 2 diabetes

**DOI:** 10.3389/fcvm.2023.1086978

**Published:** 2023-01-30

**Authors:** Tingting Wang, Jiani Xu, Hong Zhang, Lichan Tao, Xiaolin Huang

**Affiliations:** ^1^Department of Cardiology, The Third Affiliated Hospital of Soochow University, Changzhou, China; ^2^Department of Endocrinology, The Third Affiliated Hospital of Soochow University, Changzhou, China

**Keywords:** triglyceride-glucose index, heart failure with preserved ejection fraction, type II diabetes, insulin resistance, HbA1c - hemoglobin A1c

## Abstract

**Objectives:**

The triglyceride-glucose (TyG) index has been identified as a reliable and simple surrogate of insulin resistance. In this study, we sought to determine the association between TyG index and cardiac function among asymptomatic individuals with type 2 diabetes (T2DM) without history of any cardiovascular disease.

**Materials and methods:**

The cross-sectional study enrolled 180 T2DM patients without cardiac symptoms. Heart failure with preserved ejection fraction (HFpEF) was defined as Heart Failure Association (HFA)-PEFF score ≥ 5 points.

**Results:**

A total of 38 (21.1%) diabetic patients were identified with HFpEF. Compared with the low-TyG group (TyG index <9.47), patients in high-TyG group (TyG index ≥9.47) showed increased risk of metabolic syndrome and diastolic dysfunction (*p* < 0.05 for each). Furthermore, after adjustment of confounding variables, the TyG index showed positive correlation with risk factors of metabolic syndrome (including BMI, waist circumference, blood pressure, HbA1c, TG, TC, non-HDL-C, and fasting blood glucose, *p* < 0.05 for each) and parameters of diastolic dysfunction (E/e’ ratio, *p* < 0.0001) in patients with T2DM. Moreover, Receiver Operating Characteristic *curve* analysis showed that the TyG index could be better to predict the risk of suspected HFpEF than other indicators (AUC: 0.706, 95% CI: 0.612–0.801). According, on multiple regression analysis, TyG index was independently correlated with the incidence of HFpEF (odds ratio: 0.786, *p* = 0.0019), indicating that TyG index could be a reliable biomarker to predict the risk of HFpEF.

**Conclusion:**

The TyG index showed a positive correlation with the risk of subclinical HFpEF in patients with T2DM, providing a new marker to predict and treat HFpEF in diabetes.

## Introduction

Diabetes mellitus (DM) can contribute to cardiac abnormalities both structurally and functionally, predisposing individuals to a heightened risk of cardiovascular disease (CVD) (1, 2). Diabetic cardiomyopathy (DCM) was initially described as a human pathological condition in which heart failure occurred independent of coronary artery disease (CAD), hypertension, and valvular heart disease (3, 4). DCM in early stage is characterized by asymptomatic cardiac dysfunction or described as heart failure (HF) at stage B (subclinical HF). Cardiac disorders include left atrial (LA) dilatation, concentric left ventricular (LV) remodeling, LV diastolic dysfunction, and reduced global longitudinal strain (5). Regardless of concomitant LV systolic dysfunction, mounting evidence from epidemiological studies imply that comparing to healthy individuals, patients with diastolic dysfunction impart poor prognostic implications, with an increased 3-fold risk of death in diabetic patients (6). Thus, in view of the large number of diabetic patients and related cardiac complications, it will be of great importance to identify high risk individuals prone to cardiac dysfunction through effective and simple diagnostic strategy at early stage.

Clinical evidence establishes that glycemic control correlates with elevated risk of DCM (7, 8). Moreover, higher glycosylated hemoglobin, type A1c (HbA1c) variability is identified to be correlated with heightened risk of all-cause and cardiovascular mortality in diabetic patients (9). However, the severity and progression of HF vary in diabetic patients with poor glycemic control, and HF may also occur in patients with well-controlled blood glucose levels. Therefore, in addition to hyperglycemia, other risk factors may also participate in the development of clinical manifestation of DCM.

Chronic hyperglycemia and insulin resistance (IR) are the major mechanisms involved in the pathology of diabetic complications (10). During diabetic and IR states, metabolic, structural, and functional alterations in the myocardium and vascular beds or vascular tissues lead to coronary artery disease (CAD), myocardial ischemia, and HF (5). Previous clinical studies demonstrated that homeostasis model of IR (HOMA-IR) was independently correlated with LV diastolic dysfunction (11). However, it is not clear whether IR predicts subclinical cardiac diastolic dysfunction in patients with diabetes.

At present, no specific methods are available for the accurate detection of IR. HOMA-IR is a validated and widely used surrogate by incorporating insulin concentrations and serum glucose level, but the clinical practice is limited due to atypical assessment of serum insulin levels (12). The triglyceride glucose (TyG) index, a product derived from fasting triglycerides (TG) and fasting blood pressure (FBG), has been proven to be superior to HOMA-IR in evaluating IR in individuals with or without diabetes (13). Therefore, the aim of this study was to investigate the association between TyG index and the risk of cardiac diastolic dysfunction in patients with type 2 diabetes (T2DM) and the predictive value of TyG index to provide novel clues for the early recognition and prevention of HF in diabetic patients.

## Methods

### Subject design and recruitment

A retrospective consecutive case series of T2DM patients hospitalized in the Department of Endocrinology at the Changzhou First People’s Hospital (Changzhou, Jiangsu, China) were recruited from April 2018 to May 2022. The Inclusion criteria were: (1) diagnosed T2DM according to the criteria of World Health Organization (14) and Chinese Diabetes Society (15) without cardiac symptoms; (2) aged from 18 to 70 years old independent of T2DM duration. The exclusion criteria were: (1) subjects with hypertension (Hypertension was diagnosed according to the following Chinese hypertension guidelines: a mean systolic blood pressure ≥ 140 mmHg and/or a mean diastolic blood pressure ≥ 90 mmHg and/or self-reported use of antihypertensive medication in the past 2 weeks) (16, 17), CAD, atrial fibrillation, structural heart disease or history of any cardiovascular-related disease; (2) subjects with diabetic complications including macro and microvascular diseases such as neuropathy, retinopathy, kidney disease, stroke and peripheral vascular disease; (3) pregnancy; (4) other serious comorbidities, including thyroid disturbances, malignant tumors, liver and renal insufficiency, rheumatic diseases or major mental illness. All the subjects signed written informed consent forms before the start of this study. The study was approved by the Institutional Review Committee and the Ethics Committee of the Third Affiliated Hospital of Soochow University.

### Clinical and biochemical measurements

Baseline characteristics including age, sex, weight, height, body mass index (BMI), waist circumference, diabetic duration and other complete medical history were recorded in detail on the day of admission. After fasting for at least 8 h, peripheral venous blood was collected before administration of hypoglycemic drugs on the morning after admission. Briefly, the concentration of HbA1c was evaluated through high performance liquid chromatography. Glutamic oxaloacetic transaminase (AST), alanine aminotransferase (ALT), creatinine (Cr), blood urea nitrogen (BUN), homocysteine, total cholesterol (TC), TG, low-density lipoprotein cholesterol (LDL-C), high-density lipoprotein cholesterol (HDL-C), FBG, and C peptide (0 min, 30 min, 60 min, 120 min, and 180 min) were analyzed by an automatic analyzer. Blood pressure including systolic (SBP) and diastolic blood pressure (DBP) were measured three times at 2-min intervals following at least 5 min of rest on the morning after admission.

### Echocardiographic measurements

The following parameters were measured and analyzed by echocardiography: the left atrial diameter (LAD), left ventricular end-systolic diameter (LVESD), left ventricular end-diastolic diameter (LVEDD), interventricular septal diameter (IVSD), left ventricular posterior wall thickness (LVPWT), left ventricular ejection fraction% (LVEF%), peak late diastolic trans-mitral flow velocity (MFV A), peak early diastolic trans-mitral flow velocity (MFV E), mitral valve septal velocity e, mitral valve lateral velocity e, and LA volume. e’ was defined as: (ventricular septal velocity e + mitral valve velocity e)/2. Left atrial volume index (LAVI) was defined as: LA volume/body surface area, where the body surface area was equal to 0.0128*weight (kg) + 0.006*height (cm) - 0.1529. Left ventricular mass index (LVMI) was defined as: 0.8*10.4 (IVSD+LVPWT+LVEDD). Relative ventricular wall thickness (RWT) was defined as: (LVPWT/LVEDD) *2.

### TyG index and HFA-PEFF score definition

The TyG index was calculated as: In [fasting TG (mg/dl) x fasting glucose (mg/dl)/2]. The Heart Failure Association (HFA)-PEFF score was originated from HFA-PEFF diagnostic algorithm, including functional, morphological, and biomarker domains (18). Data of the peak tricuspid velocity and global longitudinal strain were not available in this study. In the HFA-PEFF diagnostic algorithm, a total score ≥ 5 points was identified to be diagnostic of heart failure with preserved ejection fraction (HFpEF), while a score ≤ 1 was considered to be very unlikely of HFpEF. Patients with an intermediate PEFF score (2–4 points) required further functional and etiology assessment (18).

### Statistical analysis

All data in this study were analyzed using SPSS statistical software 26.0. *p* value <0.05 was defined to be of statistical significance. The specific statistical analysis in this study were outlined as follows.

#### Baseline and echocardiographic data of subjects

The baseline and echocardiographic data of diabetic patients were stratified based on binary TyG index. The differences between two groups were evaluated, continuous normal distribution variables were expressed as mean ± standard deviation (SD) by independent sample t-test, nonnormal distribution variables were expressed as median P50 (P25, P75) by Mann–Whitney U test, and the categorical variables were presented as number (percentage) and analyzed by *χ*^2^ test.

#### Correlation analysis

Pearson correlation analysis was used to analysis independent variables with the TyG index. Partial correlation analysis was used to correct suspicious confounding factors (make it/them a constant).

#### Logistic regression

A logistic multivariable regression analysis with cardiac diastolic dysfunction categorized as HFA-PEFF score (≤1, 2–4, and ≥ 5 points) was used to determine the associations between the TyG index and HFpEF. The goodness of fit of the regression model was assessed by Hosmer-Lemeshow test (*p* > 0.05). In the logistic regression analysis, three models were set up, Model 1: adjusted by age and sex; Model 2: adjusted by BMI, waist circumference, and diabetic duration based on model 1; Model 3: adjusted by estimated glomerular filtration rate (eGFR), mean arterial pressure (MAP), TC and HbA1c based on model 2.

#### Receiver operating characteristic curve analysis

Receiver operating characteristic curve (ROC) analysis was constructed to evaluate the predictive value of TyG index, FBG, postprandial blood glucose (PBG), TG, TC, LDL-C, TG/HDL-C, and HbA1c for the subclinical HFpEF presence according to the value of the area under the ROC curve (AUC).

#### Subgroup analysis

A stratified analysis was conducted based on sex, age, HbA1c and T2DM duration to eliminate the interference of confounding factors. Among them, means of age and HbA1c were defined as stratification criteria while the median of duration was used for the cut-off point since the latter does not conform to the normal distribution.

## Results

### Clinical characteristics of T2DM patients stratified by binary TyG index

A total of 180 subjects with T2DM (102 men and 78 women), aged 53.82 ± 9.20 years old, with a median diabetic duration of 6 years (interquartile range 0.75–10 years) were included in this study. According to the mean value of TyG index, diabetic patients were separated into two groups as low-TyG group (TyG index <9.47, *N* = 88) and high-TyG group (TyG index ≥9.47, *N* = 92). Compared with the low-TyG group, patients in high-TyG group showed higher levels of metabolic syndrome-related risk factors, as indicated by elevated BMI, waist circumference, SBP, DBP, HbA1c, TG, TC, non-HDL-C, and FBG, and reduced HDL-C (*p* < 0.05 for each; Table 1). Accordingly, the TyG index was positively associated with these metabolic parameters (including BMI, waist circumference, MAP, SBP, DBP, TG, TC, non-HDL, LDL-C, and FBG; *p* < 0.05 for each) and negatively associated with HDL-C and eGFR (*p* < 0.05; Supplementary Table S1) after adjusting for age, sex, and duration of diabetes. In addition, Patients in high-TyG group were more likely to use biguanides, glucagon-like peptide 1 (GLP-1) receptor agonists, and statins (*p* < 0.05 for each; Table 1).

**Table 1:** Clinical and metabolic characteristics in T2DM patients stratified by binary TyG index.

Variables	TyG index <9.47 (*N* = 88)	TyG index ≥9.47(*N* = 92)	*p*-value
Age (years)	54.06 ± 9.62	53.80 ± 8.63	0.9854
Male (*n*, %)	52 (59.1%)	50 (54.3%)	0.5209
Diabetes duration(years)	6.00 (0.50–10.00)	6.00 (1.00–10.00)	0.5549
BMI (kg/m^2^)	23.62 ± 3.36	25.22 ± 3.41	0.0013
Waist circumference (cm)	86.95 ± 8.70	90.64 ± 9.94	0.0133
MAP (mmHg)	91.14 ± 9.86	94.57 ± 10.09	0.0224
SBP (mmHg)	122.81 ± 12.70	125.63 ± 12.82	0.0491
DBP (mmHg)	75.79 ± 9.65	78.87 ± 10.08	0.0308
HbA1c (%)	9.00 (7.30–10.70)	10.15 (8.53–11.75)	0.0094
ALT (U/L)	18.00 (12.30–25.70)	22.00 (14.03–32.18)	0.0839
AST (U/L)	18.00 (15.30–23.00)	20.00 (16.00–25.60)	0.1884
TG (mmol/L)	1.29 ± 0.47	3.41 ± 2.65	<0.0001
TC (mmol/L)	4.60 ± 0.98	5.16 ± 1.15	0.0005
HDL-C (mmol/L)	1.18 ± 0.30	0.91 ± 0.19	<0.0001
Non-HDL (mmol/L)	3.41 ± 0.90	4.25 ± 1.15	<0.0001
LDL-C (mmol/L)	2.66 ± 0.79	2.89 ± 0.82	0.0593
Serum creatinine (μmol/L)	59.26 ± 14.06	62.21 ± 16.13	0.1937
eGFR [mL/(min*1.73m^2^)]	114.28 (99.21–145.34)	112.18 (92.61–135.29)	0.2787
FBG (mmol/L)	6.80 (5.79–8.79)	10.02 (7.83–12.82)	<0.0001
Postprandial glucose (mmol/L)	13.67 (10.20–16.86)	14.65 (11.85–17.45)	0.0770
Homocysteine (umol/L)	10.04 (8.63–10.68)	9.98 (9.70–10.80)	0.4915
BNP (pg/mL)	29.00 (18.88–43.33)	28.75 (15.25–49.98)	0.9282
cTnI (ng/mL)	0.0021 (0.0015–0.0037)	0.0019 (0.0010–0.0035)	0.1226
CK-MB (U/L)	1.56 (1.00–1.71)	1.30 (0.80–1.71)	0.0885
Myo (ng/mL)	20.17 (13.73–23.50)	18.75 (12.45–25.80)	0.7053
Medication			
Insulin (n, %)	62 (70.5%)	72 (78.3%)	0.2300
Biguanides (n, %)	61 (69.3%)	79 (85.9%)	0.0076
Sulfonylureas (n, %)	13 (14.8%)	12 (13.0%)	0.7374
α-glucosidase inhibitors (n, %)	62 (70.5%)	68 (73.9%)	0.6046
Thiazolidinediones (n, %)	2 (2.3%)	3 (3.3%)	1.0000
SGLT2 inhibitors (n, %)	23 (26.1%)	24 (26.1%)	0.9940
GLP-1 receptor agonists (n, %)	11 (12.5%)	4 (4.3%)	0.0479
DPP4 inhibitors (n, %)	18 (20.5%)	24 (26.1%)	0.3718
Statins (n, %)	43 (48.9%)	59 (64.1%)	0.0388

BMI, body mass index; MAP, mean arterial pressure; SBP, Systolic blood pressure; DBP, Diastolic blood pressure; HbA1c, glycosylated hemoglobin, type A1c; Glutamic oxaloacetic transaminase (AST), alanine aminotransferase (ALT); TG, triglycerides; TC, total cholesterol; HDL-C, high-density lipoprotein cholesterol; LDL-C, low-density lipoprotein cholesterol; eGFR, estimated glomerular filtration rate; FBG, fasting blood glucose; BNP, brain natriuretic peptide; cTnI, cardiac tropin I; CK-MB, creatine Kinase-MB; Myo, myoglobin; SGLT2 inhibitors, sodium-glucose cotransporter 2 inhibitors; GLP-1 receptor agonists, glucagon-like peptide 1 receptor agonists; dipeptidyl peptidase-4 (DPP4) inhibitors, dipeptidyl peptidase 4 inhibitors.

### Echocardiographic characteristics of T2DM patients stratified by binary TyG index

Compared with patients in low-TyG group, patients in high-TyG group showed cardiac diastolic disorder, as exhibited by elevated E/e’ ratio and LA volume (*p* < 0.05 for each; Table 2). Additionally, correlation analysis showed that the TyG index was positively associated with E/e’ ratio (*r* = 0.273, *p* = 0.0002) and negatively associated with septal e’ velocity (*r* = −0.245, *p* = 0.0010) and lateral e’ velocity (*r* = −0.339, *p* < 0.0001; Supplementary Table S2) after adjusting for age, sex, and duration of diabetes. However, no differences were detected in the systolic function and ventricular remodeling between two groups.

**Table 2:** Echocardiographic data of in T2DM patients stratified by binary TyG index.

Variables	TyG index <9.47 (*N* = 88)	TyG index ≥9.47 (*N* = 92)	*p*-value
LVEF (%)	64.44 ± 2.80	63.99 ± 2.95	0.0905
LVEDD (mm)	46.53 ± 3.98	47.30 ± 3.40	0.1636
LVESD (mm)	30.15 ± 2.78	30.74 ± 2.43	0.1295
IVSD (mm)	9.00 (8.00–9.00)	9.00 (8.00–9.00)	0.1583
LVPWT (mm)	9.00 (8.00–9.00)	9.00 (8.00–9.00)	0.1751
septal e (cm/s)	7.30 (6.00–8.40)	7.00 (5.63–8.00)	0.0609
lateral e (cm/s)	10.00 (8.60–11.08)	8.55 (7.53–10.10)	0.0031
E/A	0.85 (0.74–1.08)	0.87 (0.75–1.11)	0.6075
E/e’	8.23 (6.92–10.37)	9.83 (8.22–11.05)	0.0015
LA (mm)	34.40 ± 3.63	34.93 ± 4.15	0.3578
LA volume (mm)	43.67 ± 11.31	47.84 ± 12.72	0.0215
LAVI (mL/m^2^)	26.57 ± 6.65	28.00 ± 7.89	0.1899

LVEF (%), left ventricular ejection fraction%; LVEDD, left ventricular end-diastolic diameter; LVESD, left ventricular end-systolic diameter; IVSD, interventricular septal diameter; LVPWT, left ventricular posterior wall thickness; LA, left atrial; LAVI, left atrial volume index.

### ROC analysis for the identification of diabetic patients with risk of HFpEF

To confirm that TyG index is particularly well related to IR in patients with diabetes, we evaluated the association between other simultaneously measured IR or insulin sensitivity indices and TyG index. Previous studies have revealed that the lipid profile in T2DM patients with IR often manifested as a TG/HDL-C axis disorder, with elevations of TG and reductions of HDL-C. TG/HDL-C ratio was thus identified as one of the major risk factors for IR and CVD (19). Additionally, C-peptide is secreted from pancreatic β cells at an equimolar ratio to insulin, reflecting endogenous insulin secretion (20). In this study, patients with a higher TyG index had a higher TG/HDL-C ratio and C-peptide values at 0 min and 30 min (Table 3; *p* < 0.05 for each), suggesting that TyG index could be a reliable marker for IR in T2DM patients.

**Table 3:** Data of insulin resistance indices in T2DM patients stratified by binary TyG index.

Variables	TyG index <9.47 (*N* = 88)	TyG index ≥9.47 (*N* = 92)	*p*-value
TG/HDL-C	1.04 ± 0.49	3.75 ± 4.42	<0.0001
C peptide 0 min (pmol/L)	452.26 ± 256.93	637.63 ± 285.30	<0.0001
C peptide 30 min (pmol/L)	636.67 ± 351.08	798.57 ± 350.84	0.0019
C peptide 60 min (pmol/L)	861.46 ± 494.17	989.97 ± 457.90	0.0545
C peptide 120 min (pmol/L)	1121.06 ± 705.74	1302.37 ± 727.72	0.0762
C peptide 180 min (pmol/L)	1041.36 ± 588.20	1151.46 ± 633.69	0.2480

TG, triglycerides; HDL-C, high-density lipoprotein cholesterol.

**Figure 1:**
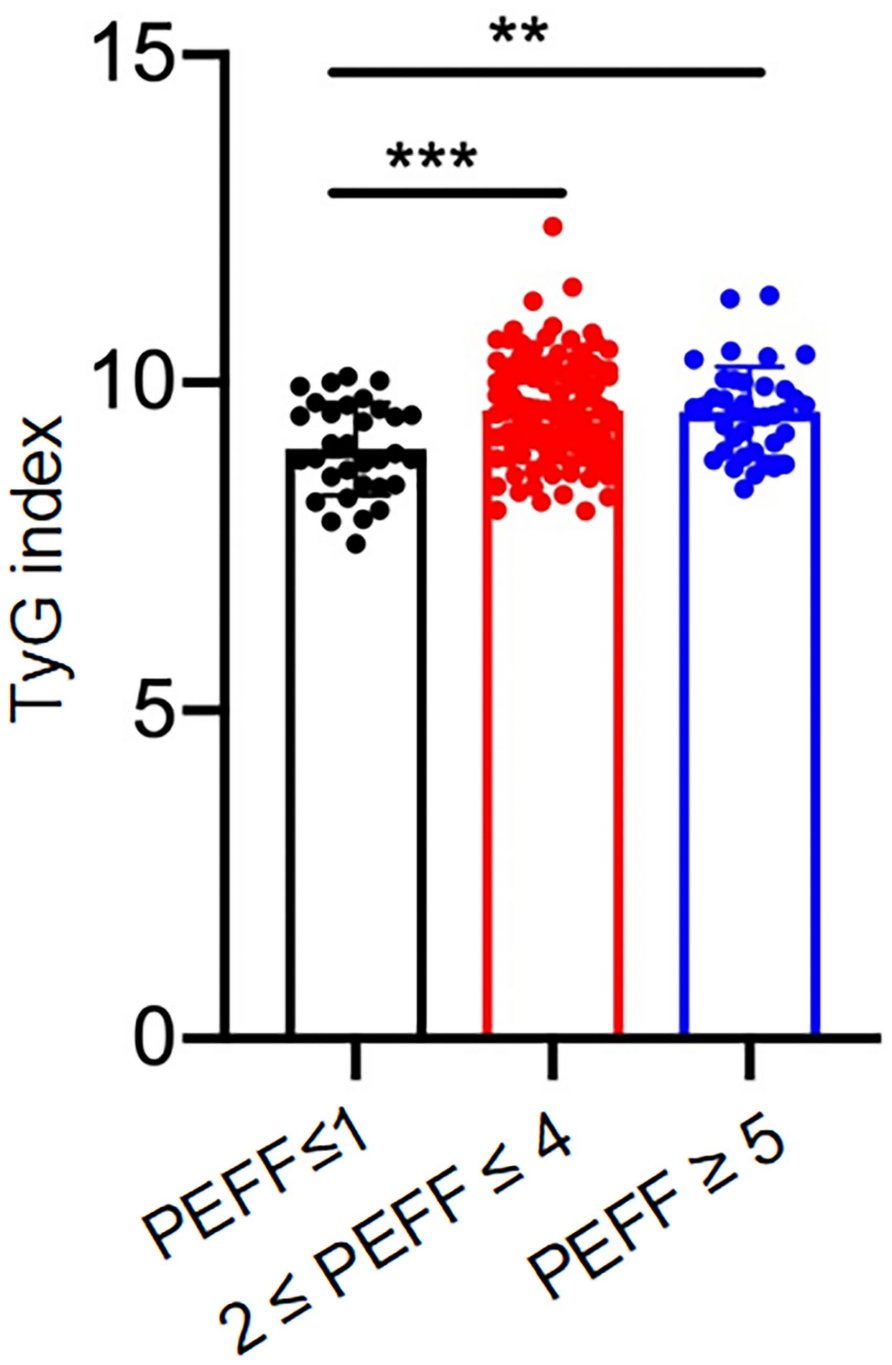
TyG index in diabetic patients stratified by HFA-PEFF score. **p < 0.05. ***p < 0.001.

**Figure 2:**
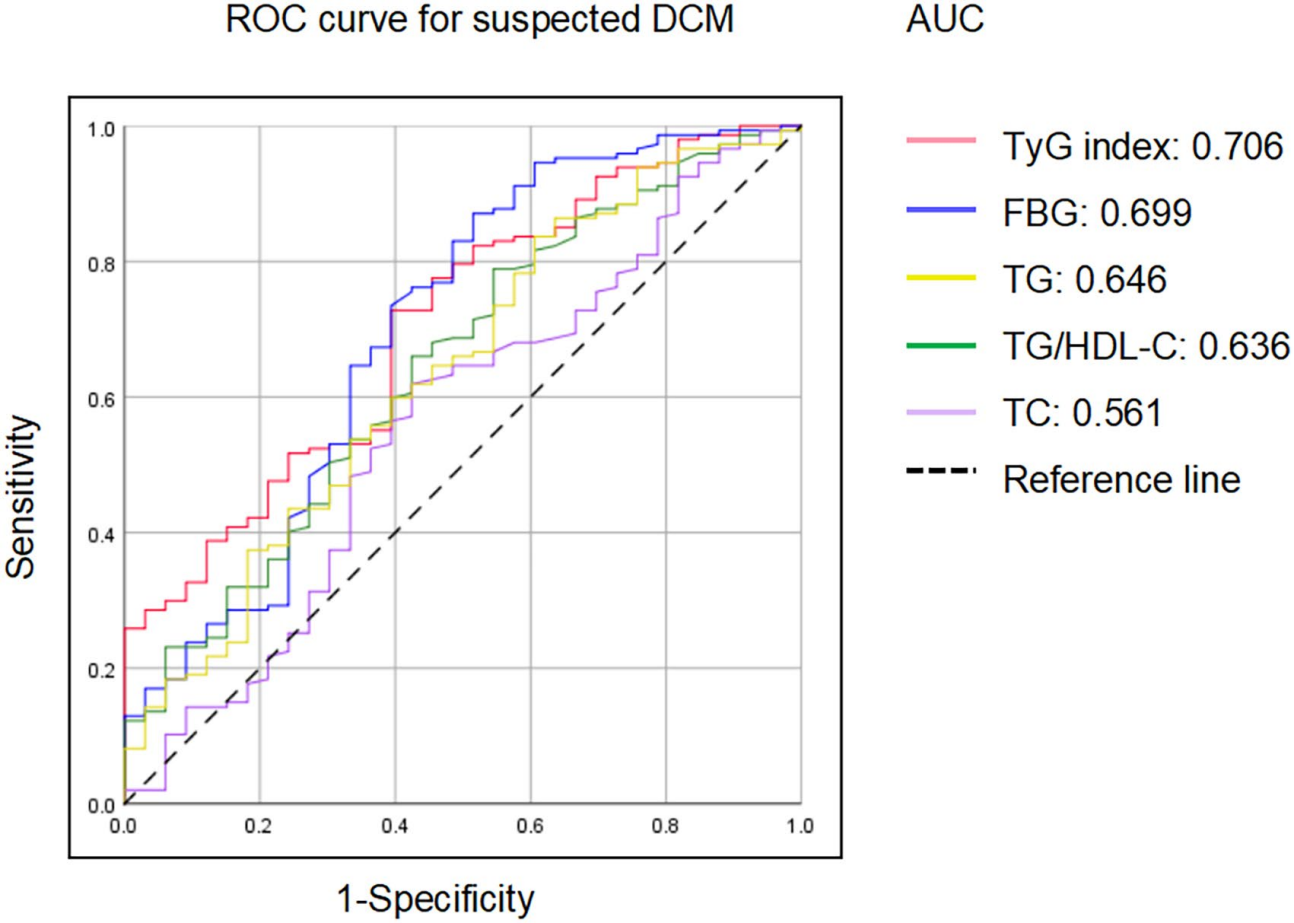
ROC analysis for the identification of diabetic patients with risk of HFpEF. TyG, triglyceride-glucose; FBG, fasting blood glucose; TG, triglycerides; HDL-C, high-density lipoprotein cholesterol; TC, total cholesterol.

Next, we compared the significance of TyG index, TG/HDL-C ratio, FBG, and HbA1c to identify diabetic patients with subclinical cardiac dysfunction. First, previous studies have demonstrated that DM-related HF shifted from an asymptomatic stage to HFpEF, which was manifested by LV shrinkage but not LV dilatation, and finally developed to LV dilatation with reduced EF (HFrEF) (21). The HFA-PEFF score was a scoring system for suspected HFpEF assessing brain natriuretic peptide (BNP) and echocardiographic parameters (18). In this study, among 180 asymptomatic T2DM patients, 38 (21.1%) patients were identified with HFpEF as calculated by HFA-PEFF score ≥ 5 points, 33 (18,3%) patients were identified negative (HFA-PEFF score ≤ 1), and 109 (60.6%) patients were suspected to be positive (2 ≤ HFA-PEFF score ≤ 4). Compared to the negative group, the TyG index were higher in suspicious positive and positive HFpEF group (Figure 1). Furthermore, ROC analysis for detecting suspicious or positive HFpEF showed that AUC of TyG index was 0.706 (95% confidence interval (CI): 0.612–0.801), significantly higher than that of FBG, PBG, TG, TC, LDL-C, TG/HDL-C, and HbA1c (AUC < 0.5 not shown in the Figure 2). When the Youden Index reached the maximum, the optimal cut-off point of the TyG index was 9.0067. The corresponding sensitivity and specificity were 72.8 and 60.6%, respectively.

### Multivariate analysis of the correlation between TyG index and HFA-PEFF score in diabetic patients

To further explore the relationship between TyG index and HFpEF in diabetic patients, multivariate logistic stepwise regression analysis was performed. Data showed that the TyG index was independently correlated with the risk of HFpEF (HFA-PEFF score ≥ 5) after adjusting for age, sex, BMI, waist circumference, MAP, diabetes duration, TC, eGFR, and HbA1c (Odds Ratio (OR): 0.786, 95% CI: 0.290–1.282, *p* = 0.0019; Table 4). Then we performed subgroup analyzes to evaluate the impact of other risk factors based on the following stratification variables: sex, age, HbA1c, and duration of diabetes (Table 5). An increased TyG index remained significantly correlated with the risk of HFpEF in the subgroups of age, sex, HbA1c, and duration of diabetes (*p* < 0.05 for each). Stronger correlations were found in the subgroups of male (OR: 0.877, *p* = 0.0174), age < 54 years (OR: 1.055, *p* = 0.0078), HbA1c ≥ 9.75% (OR: 1.084, *p* = 0.0032) and duration of diabetes after multivariable adjustment. However, no significant association was detected in female patients, patients aged ≥54, and patients with HbA1c < 9.75%. Clinical and metabolic characteristics stratified by HbA1c, and duration of DM were presented in Supplementary Tables S3 and S4, respectively.

**Table 4:** Logistic regression analysis of the association between the TyG index and HFA-PEFF score.

	EXP (95%CI)	*p*-value
Model 1	0.626 (0.226–1.025)	0.0021
Model 2	0.640 (0.219–1.062)	0.0029
Model 3	0.786 (0.290–1.282)	0.0019

Model 1, Adjusted for age, sex; Model 2, Model 1+ BMI, Waist circumference, DM duration; Model 3, Model 2+ eGFR, MAP, TC, HbA1c. BMI, body mass index; eGFR, estimated glomerular filtration rate; MAP, mean arterial pressure; TC, total cholesterol; HbA1c, glycosylated hemoglobin, type A1c.

**Table 5:** Subgroup analysis of odds ratios of the TyG index with HFA-PEFF score in T2DM.

	N	Model 1		Model 2		Model 3		OR (95%CI)	*p*-value	OR (95%CI)	*p*-value	OR (95%CI)	*p*-value
Sex							
Female	78	0.574 (−0.016–1.164)	0.0565	0.730 (0.094–1.366)	0.0246	0.542 (−0.216–1.300)	0.1608
Male	102	0.684 (0.131–1.238)	0.0154	0.521 (−0.085–1.128)	0.0918	0.877 (0.154–1.599)	0.0174
Age (year)							
<54	78	0.696 (0.118–1.274)	0.0182	0.742 (0.124–1.361)	0.0187	1.055 (0.277–1.832)	0.0078
≥54	102	0.564 (−0.014–1.142)	0.0558	0.566 (−0.047–1.178)	0.0701	0.628 (−0.060–1.316)	0.0736
HbA1c (%)							
<9.75%	88	0.573 (−0.041–1.189)	0.0677	0.447 (−0.211–1.104)	0.1827	0.322 (−0.470–1.135)	0.4167
≥9.75%	92	0.674 (0.139–1.208)	0.0135	0.781 (0.203–1.359)	0.0081	1.084 (0.363–1.805)	0.0032
Duration (year)							
<6	85	0.647 (0.079–1.216)	0.0255	0.584 (−0.056–1.225)	0.0738	1.008 (0.215–1.802)	0.0127
≥6	95	0.580 (0.005–1.155)	0.0481	0.634 (0.041–1.226)	0.0360	0.752 (0.002–1.502)	0.0494

Model 1, Adjusted for age, sex; Model 2, Model 1+ BMI, Waist circumference, DM duration; Model 3, Model 2+ eGFR, MAP, TC, HbA1c.

## Discussion

This retrospective study demonstrated that among 180 asymptomatic patients with T2DM, 38 (21.1%) patients were identified with HFpEF as calculated by HFA-PEFF score ≥ 5 points. Elevated TyG index was positively corrected with metabolic syndrome-related risk factors (BMI, waist circumference, blood pressure, HbA1c, TG, TC, non-HDL-C, and FBG, *p* < 0.05 for each) and parameters of diastolic dysfunction (E/e’ ratio, *p* < 0.0001) after adjustment of confounding factors. Importantly, TyG index was independently correlated with greater risk of developing HFpEF as evaluated by HFA-PEFF. Our results suggested that in diabetic patients, TyG index might be considered as a reliable biomarker to identify asymptomatic patients with high HFpEF risk.

The TyG index, derived from FBG and TG, was proven to be a reliable and simple surrogate for metabolic syndrome and IR (22). Mounting evidence has proved the crucial value of TyG index in predicting diabetic complications in patients with T2DM (23–25). Study by Liu et al. showed a significant association between TyG index and the risk of diabetic nephropathy in 682 adult patients with T2DM (26). Pan et al. confirmed the predictive value of TyG index in distinguishing diabetic patients at an increased risk of lower limb vascular stenosis and nephric microvascular disorder (18). Furthermore, recent studies suggested that TyG index could be recognized as a risk factor for CVD even in asymptomatic patients. Lee et al. showed that higher level of TyG index was correlated with increased risk of coronary artery stenoses (CAS) in asymptomatic diabetic patients (27). Thai et al. confirmed this hypothesis and proposed that TyG index was positively associated with the number and severity of artery stenoses (28). However, the predictive value of TyG index in subclinical HF in diabetic patients has not been well evaluated. In accordance with prior studies, our findings showed that the TyG index had a strong association with metabolic syndrome and HFpEF in subjects with T2DM, including BMI, waist circumference, blood pressure, HbA1c, TG, TC, HDL-C, non-HDL-C, LDL-C and FBG.

As an indicator of IR, the relationship between TyG index and the occurrence of CVD in different groups, including non-diabetic and diabetic individuals, has been widely explored (22). However, few studies have investigated the association between TyG index and cardiac structure and hemodynamics evaluated by echocardiography, which may predict the risk of CVD. An observational study enrolled 823 general subjects found that high TyG index was correlated with elevated LA diameter and decreased LVEF (%) and ankle-branchial index (ABI) (29). These results were partly consistent with the data by Wang et al., in which TyG index was positively associated with cardiac hemodynamics such as LVESD, LVEDV, LVPW, IVS, and LV mass and negatively associated with LVEF. The latter study was conducted in 201 healthy controls and 446 asymptomatic patients with T2DM (30). Nevertheless, our results demonstrated that TyG index was positively correlated with E/e’ ratio and negatively correlated with septal e’ and lateral e’, but not correlated with parameters of cardiac systolic function. Of note, high TyG index was significantly positively correlated with increased risk of HFA-PEFF score ≥ 5 points, indicating a strong association between the TyG index and cardiac diastolic function. This inconsistence may be attributed to different recruited subjects, diverse diseases of enrolled population, and the potential impacts of drugs. Our study focused on exploring the diagnostic value of the TyG index to early detection of cardiac structural changes in diabetic patients, which may be of special significance for clinical cardiovascular risk assessment and secondary prevention.

Moreover, previous studies showed that myocardial dilatation defects are reported to be abnormal in patients with hyperglycemia and IR. Thus, the factors that determine TyG levels (high TG and high glucose at the baseline condition) related to the following conditions (1) hypo-insulinemia with hyperglycemia and (2) hyperinsulinemia and hyperglycemia with insulin resistance. To clarify the relationship between these conditions, we evaluated the association between TyG index and IR, or insulin deficiency. Our results showed that TyG index was positively related to HG/HDL-C value (IR biomarker) and C-peptide at 0 min and 30 min (insulin secretion marker). Additionally, subgroup analysis demonstrated that there was stronger association between TyG index and increased risk of HFpEF in patients with insufficient glycemic control (HbA1c ≥ 9.75%), suggesting that TyG index was mainly dependent on the second condition. Importantly, ROC analysis revealed that compared to sustained hyperglycemia status, TyG index preserved a higher predictive value for HFpEF in patients with T2DM, confirming the crucial role of IR in diabetic related cardiac dysfunction.

There are some limitations need to be emphasized in this study. Firstly, parameters evaluating cardiac diastolic function and HFA-PEFF score were incomplete, including tricuspid valve velocity and global longitudinal strain data. These missing data in the HFA-PEFF score may reduce statistical power and cause selection bias. More complete echocardiographic data in the future may improve the reliability and stability of TyG index in predicting diabetic patients with high HF risk. Secondly, HF is a series of dynamic and progressive disorders, the calculation of the baseline TyG index alone does not represent the longitudinal correlation between the TyG index and risk of diabetes-induced HF over time. Cumulative TyG index (the summation of average TyG index for each pair of consecutive assessments multiplied by the time between these two-consecutive inclusion in years) may be better than single TyG index at baseline in predicting HF or even other CVDs (31). Finally, the number of eligible patients was relatively limited, which may be due to the very specific population in this study. DM is often accompanied by different subtypes of CVDs involving multiple risk factors. Nevertheless, clinical studies of diabetic status itself (hyperglycemia with or without IR) on CVDs, especially on subclinical CVDs are limited. Thus, to simply the impact of diabetes on cardiac structure and function, we screened diabetic patients without any other risk factors to confirm the predictive value of TyG index in subclinical HF. Therefore, more sample size and multi-center studies are warranted to explore the crucial role of hyperglycemia and IR in diabetic complications. The diagnostic criteria for subclinical diabetic cardiac dysfunction also needs to be further refined.

## Conclusion

In conclusion, we explored a significant correlation between TyG index and an increased risk of HFpEF in asymptomatic patients with T2DM. IR plays a crucial role in the pathophysiology of HFpEF and may be identified as a novel target for its prevention and treatment. Further studies are warranted to explore the correlation between the IR parameters, especially TyG index, and the risk of HF in patients with T2DM at different stages.

## Data availability statement

The raw data supporting the conclusions of this article will be made available by the authors, without undue reservation.

## Ethics statement

The studies involving human participants were reviewed and approved by The Third Affiliated Hospital of Soochow University. The patients/participants provided their written informed consent to participate in this study.

## Author contributions

LT and XH designed the work. TW wrote the original version of this manuscript and analyzed the clinical data. LT performed the manuscript reviewing and editing. JX and HZ collected the clinical data. All authors read and approved the final manuscript.

## Funding

This research was funded by the National Natural Science Foundation of China (NSFC) grants 82170356 and Changzhou Sci&Tech Program grant CJ20210091.

## Conflict of interest

The authors declare that the research was conducted in the absence of any commercial or financial relationships that could be construed as a potential conflict of interest.

## Publisher’s note

All claims expressed in this article are solely those of the authors and do not necessarily represent those of their affiliated organizations, or those of the publisher, the editors and the reviewers. Any product that may be evaluated in this article, or claim that may be made by its manufacturer, is not guaranteed or endorsed by the publisher.

## Abbreviations

TyG: triglyceride-glucose
T2DM: type 2 diabetes
HFpEF: Heart failure with preserved ejection fraction
HFA: Heart Failure Association
ROC: receive operating characteristic
OR: odds ratio
CI: confidence interval
DM: Diabetes mellitus
CVD: cardiovascular disease
HF: heart failure
LA: left atrial
LV: left ventricular
HbA1c: Hemoglobin type A1c.
IR: insulin resistance
CAD: coronary artery disease
HOMA-IR: homeostasis model of insulin resistance
MAP: mean arterial pressure
SBP: systolic blood pressure
DBP: diastolic blood pressure
TG: triglycerides
ALT: glutamic oxaloacetic transaminase
AST: alanine aminotransferase
Cr: creatinine
BUN: blood urea nitrogen
TC: total cholesterol
LDL-C: low-density lipoprotein cholesterol
HDL-C: high-density lipoprotein cholesterol
FBG: fasting blood glucose
eGFR: estimated glomerular filtration rate
LAD: left atrial diameter
LVESD: left ventricular end-systolic diameter
LVEDD: left ventricular end-diastolic diameter
IVSD: interventricular septal diameter
LVPWT: left ventricular posterior wall thickness
LVEF: left ventricular ejection fraction
MFV A: peak late diastolic trans-mitral flow velocity
MFV E: peak early diastolic trans-mitral flow velocity
LVMI: Left ventricular mass index
RWT: Relative ventricular wall thickness
SD: standard deviation
AUC: area under curve
CAS: coronary artery stenoses
ABI: ankle-branchial index
