# Supplementary material for: Triglyceride-glucose index for the detection of subclinical heart failure with preserved ejection fraction in patients with type 2 diabetes
Source: Front Cardiovasc Med. 2023 Jan 30;10:1086978. doi: 10.3389/fcvm.2023.1086978 (PMC9923050; doi:10.3389/fcvm.2023.1086978)
Supplement: Supplementary file 1 [file Table_1.docx]

Table S1. Correlation between TyG index and metabolic parameters.

| Variables | r | *P*-value | Adjusted r | Adjusted *P*-value |
| --- | --- | --- | --- | --- |
| BMI (kg/m^2^) | 0.284 | <0.0001 | 0.291 | <0.0001 |
| Waist circumference (cm) | 0.211 | 0.0046 | 0.227 | 0.0024 |
| HbA1c (%) | 0.140 | 0.0602 | 0.144 | 0.0545 |
| MAP (mmHg) | 0.228 | 0.0020 | 0.240 | 0.0013 |
| SBP (mmHg) | 0.210 | 0.0046 | 0.213 | 0.0044 |
| DBP (mmHg) | 0.207 | 0.0052 | 0.224 | 0.0027 |
| TG (mmol/L) | 0.740 | <0.0001 | 0.747 | <0.0001 |
| HDL-C (mmol/L) | -0.533 | <0.0001 | -0.557 | <0.0001 |
| Non-HDL (mmol/L) | 0.573 | <0.0001 | 0.579 | <0.0001 |
| LDL-C (mmol/L) | 0.106 | 0.0312 | 0.157 | 0.0374 |
| Fasting glucose (mmol/L) | 0.612 | <0.0001 | 0.607 | <0.0001 |
| Postprandial glucose (mmol/L) | 0.146 | 0.0516 | 0.125 | 0.0971 |
| eGFR (mL/(min*1.73m^2^)) | -0.173 | 0.0212 | -0.163 | 0.0286 |

Adjusted for age, sex and duration of diabetes.

TG: triglycerides; HDL-C: high-density lipoprotein cholesterol; LDL-C: low-density lipoprotein cholesterol; BNP: brain natriuretic peptide; BMI: body mass index; HbA1c: glycosylated hemoglobin, type A1c; MAP: mean arterial pressure; SBP: Systolic blood pressure; DBP: Diastolic blood pressure.

Table S2. Correlation between TyG and ventricular diastolic function

| Variables | r | *P*-value | Adjusted r | Adjusted *P*-value |
| --- | --- | --- | --- | --- |
| LVEF (%) | -0.077 | 0.3051 | -0.074 | 0.3254 |
| LVEDD (mm) | 0.116 | 0.1198 | 0.142 | 0.0596 |
| LVESD (mm) | 0.110 | 0.1403 | 0.136 | 0.0709 |
| IVS (mm) | 0.107 | 0.1522 | 0.143 | 0.0583 |
| LVPWT (mm) | 0.097 | 0.1949 | 0.131 | 0.0824 |
| septal e (cm/s) | -0.225 | 0.0023 | -0.245 | 0.0010 |
| lateral e (cm/s) | -0.317 | <0.0001 | -0.339 | < 0.0001 |
| E/A | -0.088 | 0.2403 | -0.106 | 0.1584 |
| E/e’ | 0.284 | <0.0001 | 0.273 | 0.0002 |
| LA (mm) | 0.097 | 0.1953 | 0.118 | 0.1169 |
| LA volume (mm) | 0.167 | 0.0247 | 0.170 | 0.0239 |
| LAVI (mL/m^2^) | 0.085 | 0.2577 | 0.080 | 0.2886 |
| BNP | -0.029 | 0.7004 | -0.026 | 0.7287 |

Adjusted for age, sex and duration of diabetes.

LVEF (%): left ventricular ejection fraction%; LVEDD: left ventricular end-diastolic diameter; LVESD: left ventricular end-systolic diameter; IVSD: interventricular septal diameter; LVPWT: left ventricular posterior wall thickness; LA: left atrial; LAVI: left atrial volume index; BNP: brain natriuretic peptide.

Table S3. Clinical and metabolic characteristics in T2DM patients stratified by binary HbA1c

| Variables | HbA_1c_ < 9.75% (N=88) | HbA_1c_ ≥ 9.75% (N=92) | *P*-value |
| --- | --- | --- | --- |
| Age (year) | 55.43 ± 8.82 | 52.27 ± 9.327 | 0.0207 |
| Male (n, %) | 53 (60%) | 49 (53%) | 0.3458 |
| Diabetes duration(years) | 7.64 ± 6.09 | 6.36 ± 6.49 | 0.1750 |
| BMI (kg/m^2^) | 24.61 ± 3.21 | 24.22 ± 3.74 | 0.4589 |
| Waist circumference (cm) | 89.25 ± 8.93 | 88.75 ± 9.33 | 0.7169 |
| MAP (mmHg) | 93.33 ± 9.55 | 92.58 ± 10.64 | 0.6720 |
| SBP (mmHg) | 125.13 ± 13.19 | 122.92 ± 13.49 | 0.2702 |
| DBP (mmHg) | 77.27 ± 9.20 | 77.41 ± 10.61 | 0.9247 |
| ALT (U/L) | 19.35 (14.20-27.70) | 20.25 (12.20-28.00) | 0.4611 |
| AST (U/L) | 19.00 (16.05-25.00) | 18.85 (15.00-24.32) | 0.3109 |
| TG (mmol/L) | 1.82 (1.30-2.94) | 1.89 (1.20-2.65) | 0.6771 |
| TC (mmol/L) | 4.71 ± 0.99 | 5.06 ± 1.18 | 0.0315 |
| HDL-C (mmol/L) | 1.06 ± 0.29 | 1.03 ± 0.29 | 0.5576 |
| Non-HDL (mmol/L) | 3.65 ± 0.99 | 4.01 ± 1.19 | 0.0221 |
| LDL-C (mmol/L) | 2.67 ± 0.71 | 2.88 ± 0.90 | 0.0927 |
| Serum creatinine (µmol/L) | 63.37 ± 14.14 | 58.27 ± 15.80 | 0.0239 |
| eGFR (mL(min*1.73m^2^)) | 108.64 (90.38-131.79) | 119.58 (101.49-146.64) | 0.0193 |
| Fasting glucose (mmol/L) | 8.03 ± 2.78 | 10.20 ± 3.81 | < 0.0001 |
| Postprandial glucose (mmol/L) | 12.72 ± 3.45 | 16.07 ± 4.92 | < 0.0001 |
| Homocysteine (umol/L) | 10.46 ± 2.21 | 9.78 ± 1.68 | 0.0196 |
| BNP (pg/mL) | 28.20 (15.95-47.80) | 30.65 (18.60-43.33) | 0.4114 |
| cTnI (ng/mL) | 0.0020 (0.0011-0.0036) | 0.0020 (0.0010-0.0036) | 0.9201 |
| CK-MB (U/L) | 1.5600 (1.00-1.80) | 1.4000 (0.85-1.65) | 0.0701 |
| Myo (ng/mL) | 20.17 (14.95-30.40) | 18.45 (12.25-23.80) | 0.0296 |
| TyG index | 9.38 ± 0.73 | 9.55 ± 0.83 | 0.1385 |

BMI: body mass index; MAP: mean arterial pressure; SBP: Systolic blood pressure; DBP: Diastolic blood pressure; HbA1c: glycosylated hemoglobin type A1c; AST: glutamic oxaloacetic transaminase; ALT: alanine aminotransferase; TG: triglycerides; TC: total cholesterol; HDL-C: high-density lipoprotein cholesterol; LDL-C: low-density lipoprotein cholesterol; BNP: brain natriuretic peptide; cTnI: cardiac tropin I; CK-MB: creatine Kinase-MB; Myo: myoglobin.

Table S4. Clinical and metabolic characteristics in T2DM patients stratified by binary duration of

diabetes.

| Variables | Duration of diabetes < 6 (N=85) | Duration of diabetes ≥ 6 (N=95) | *P*-value |
| --- | --- | --- | --- |
| Age (year) | 51.12 ± 9.57 | 56.23 ± 8.17 | < 0.0001 |
| Male (n, %) | 53 (62%) | 49 (52%) | 0.1453 |
| BMI (kg/m^2^) | 24.72 ± 3.70 | 24.13 ± 3.28 | 0.2608 |
| Waist circumference (cm) | 90.53 ± 10.10 | 87.62 ± 7.94 | 0.0319 |
| MAP (mmHg) | 93.61 ± 11.31 | 92.26 ± 8.89 | 0.3700 |
| SBP (mmHg) | 123.82 ± 14.63 | 124.16 ± 12.18 | 0.8673 |
| DBP (mmHg) | 78.51 ± 10.76 | 76.31 ± 9.03 | 0.1377 |
| ALT (U/L) | 23.00 (14.00-36.00) | 18.00 (12.35-24.50) | 0.0057 |
| HbA1c (%) | 10.00 ± 2.61 | 9.59 ± 2.07 | 0.2388 |
| AST (U/L) | 20.40 (15.90-29.00) | 18.40 (16.00-21.15) | 0.0364 |
| TG (mmol/L) | 1.91 (1.14-2.91) | 1.85 (1.28-2.50) | 0.9726 |
| TC (mmol/L) | 4.82 ± 1.25 | 4.94 ± 0.96 | 0.4567 |
| HDL-C (mmol/L) | 2.76 ± 0.90 | 2.79 ± 0.74 | 0.1964 |
| Non-HDL (mmol/L) | 3.80 ± 1.29 | 3.87 ± 0.93 | 0.6836 |
| LDL-C (mmol/L) | 2.76 ± 0.90 | 2.79 ± 0.74 | 0.7920 |
| Serum creatinine (µmol/L) | 59.76 ± 16.34 | 61.67 ± 14.10 | 0.4026 |
| eGFR (mL/(min*1.73m^2^)) | 119.93 (100.48-148.26) | 110.85 (90.44-131.65) | 0.0256 |
| Fasting glucose (mmol/L) | 7.43 (6.00-10.30) | 9.00 (6.84-12.20) | 0.0027 |
| Postprandial glucose (mmol/L) | 13.78 ± 4.02 | 15.01 ± 4.97 | 0.0729 |
| Homocysteine (umol/L) | 10.26 (9.60-10.90) | 9.98 (9.25-10.26) | 0.1022 |
| BNP (pg/mL) | 28.00 (18.40-41.62) | 31.00 (17.85-49.35) | 0.4781 |
| cTnI (ng/mL) | 0.0023 (0.0015-0.0037) | 0.0019 (0.0010-0.0029) | 0.1763 |
| CK-MB (U/L) | 1.56 (1.00-1.71) | 1.50 (0.90-1.71) | 0.7762 |
| Myo (ng/mL) | 20.17 (15.70-26.60) | 17.30 (12.25-22.85) | 0.0095 |
| TyG index | 9.37 ± 0.80 | 9.56 ± 0.76 | 0.0930 |

BMI: body mass index; MAP: mean arterial pressure; SBP: Systolic blood pressure; DBP: Diastolic blood pressure; HbA1c: glycosylated hemoglobin type A1c; AST: glutamic oxaloacetic transaminase; ALT: alanine aminotransferase; TG: triglycerides; TC: total cholesterol; HDL-C: high-density lipoprotein cholesterol; LDL-C: low-density lipoprotein cholesterol; BNP: brain natriuretic peptide; cTnI: cardiac tropin I; CK-MB: creatine Kinase-MB; Myo: myoglobin.
